# miRNAs generated from Meg3-Mirg locus are downregulated during aging

**DOI:** 10.18632/aging.203208

**Published:** 2021-06-22

**Authors:** Ana-Mihaela Lupan, Evelyn-Gabriela Rusu, Mihai Bogdan Preda, Catalina Iolanda Marinescu, Cristina Ivan, Alexandrina Burlacu

**Affiliations:** 1 Laboratory of Stem Cell Biology, Institute of Cellular Biology and Pathology “Nicolae Simionescu”, Bucharest 050568, Romania; 2 Department of Experimental Therapeutics, Division of Basic Science Research, The University of Texas MD Anderson Cancer Center, Houston, TX 77030, USA

**Keywords:** aging, miRNA, Meg3-Mirg locus, cardiac fibroblasts, heart ventricles

## Abstract

Aging determines a multilevel functional decline and increases the risk for cardiovascular pathologies. MicroRNAs are recognized as fine tuners of all cellular functions, being involved in various cardiac diseases. The heart is one of the most affected organs in aged individuals, however little is known about the extent and robustness to which miRNA profiles are modulated in cardiac cells during aging. This paper provides a comprehensive characterization of the aging-associated miRNA profile in the murine cardiac fibroblasts, which are increasingly recognized for their active involvement in the cardiac physiology and pathology. Next-generation sequencing of cardiac fibroblasts isolated from young and old mice revealed that an important fraction of the miRNAs generated by the Meg3-Mirg locus was downregulated during aging. To address the specificity of this repression, four miRNAs selected as representative for this locus were further assessed in other cells and organs isolated from aged mice. The results suggested that the repression of miRNAs generated by the Meg3-Mirg locus was a general feature of aging in multiple organs. Bioinformatic analysis of the predicted target genes identified Integrin Beta-2 as an aged-upregulated gene, which was thereafter confirmed in multiple mouse organs. In conclusion, our study provides new data concerning the mechanisms of natural aging and highlights the robustness of the miRNA modulation during this process.

## INTRODUCTION

Aging is a universal, multifactorial and progressive process determined by an intrinsic decreased ability of the organism to counterbalance the adverse effects of accumulating extrinsic risk factors. It manifests as functional impairment of cells, tissues and organs and augments the susceptibility to chronic diseases. Multiple genetic and epigenetic alterations, as well as cellular disturbances in the biochemical processes have been correlated with the aging process, and part of them are currently recognized as universal hallmarks of aging [1]. MiRNAs are fine-tuners of proteostasis, having intricate roles in all cellular processes, including senescence [2] and longevity [3]. Their biogenesis involves sequential cleavages of pri-miRNAs by Drosha-DGCR8 microprocessor complex [4] and Dicer [5], which result in the synthesis of a miRNA duplex. The functional unit that promotes the miRNA-guided translation inhibition of target mRNAs is the RNA-induced silencing complex, which is comprised of a single stranded mature miRNA and Ago proteins [6, 7]. Multiple cellular factors synergize to modulate miRNA expression at transcriptional and processing levels [8] and interactions with other non-coding RNAs may alter their function [9, 10]. It is therefore conceivable that many cellular stimuli may greatly impact miRNA-mediated regulation of protein synthesis, which thus stands as an individual layer of complexity in cell signaling.

The heart is one of the most affected organs during aging, with fibrosis [11], chronic inflammation [12], hypertrophy [13] and impaired angiogenesis [14] among the major age-related manifestations [15]. When considering the structural and functional unit of the heart, the focus is usually on cardiomyocytes, even though non-myocytes form the majority of cells in the heart. Non-myocytes include multiple cell types, among which the cardiac fibroblasts, which are a heterogeneous and dynamic group of cells with important contributions to cardiac function, in healthy and diseased states. These cells (representing around 15% of the total cell number in the adult mouse myocardium) are geometrically interspersed between cardiomyocytes and are of the appropriate molecular program to allow rapid responsiveness after injury [16]. High-throughput molecular profiling of cardiac fibroblasts demonstrated the expression of a broad range of cardiogenic transcription factors, which were thought to be specific solely to the muscle compartment [17]. In corroboration, the general transcriptional profile showed that cardiac fibroblasts molecularly clustered closer to cardiomyocytes than to the fibroblasts isolated from the mouse tail [18]. These findings demonstrate that the cardiac fibroblast is a unique cell type that retains its embryological cardiac identity and has a critical role in the maintenance of the cardiac tissue.

Aging-related changes in gene expression are more pronounced in cardiac fibroblasts than in other cardiac cells (cardiomyocytes, endothelial cells, immune cells, etc.) and they mostly affect the inflammation, extracellular matrix organization and angiogenesis [19]. While miRNAs regulate different aspects of cardiovascular biology [20], little is known about the extent to which miRNA profile is modulated during aging in cardiac fibroblasts and whether the aging-associated dysregulation of miRNA profile is cell-specific or widespread.

The aims of this study were: 1) to identify relevant features of the aging-associated miRNA profile in murine cardiac fibroblasts; 2) to determine the cell and organ specificity of the aging-associated miRNA changes found in cardiac fibroblasts. This study provides evidence that aging is associated to the downregulation of miRNAs within Meg3-Mirg locus, which appears to be a common feature of many organs.

## RESULTS

### Characterization of mouse cardiac fibroblasts used for next-generation sequencing

**Figure 1:**
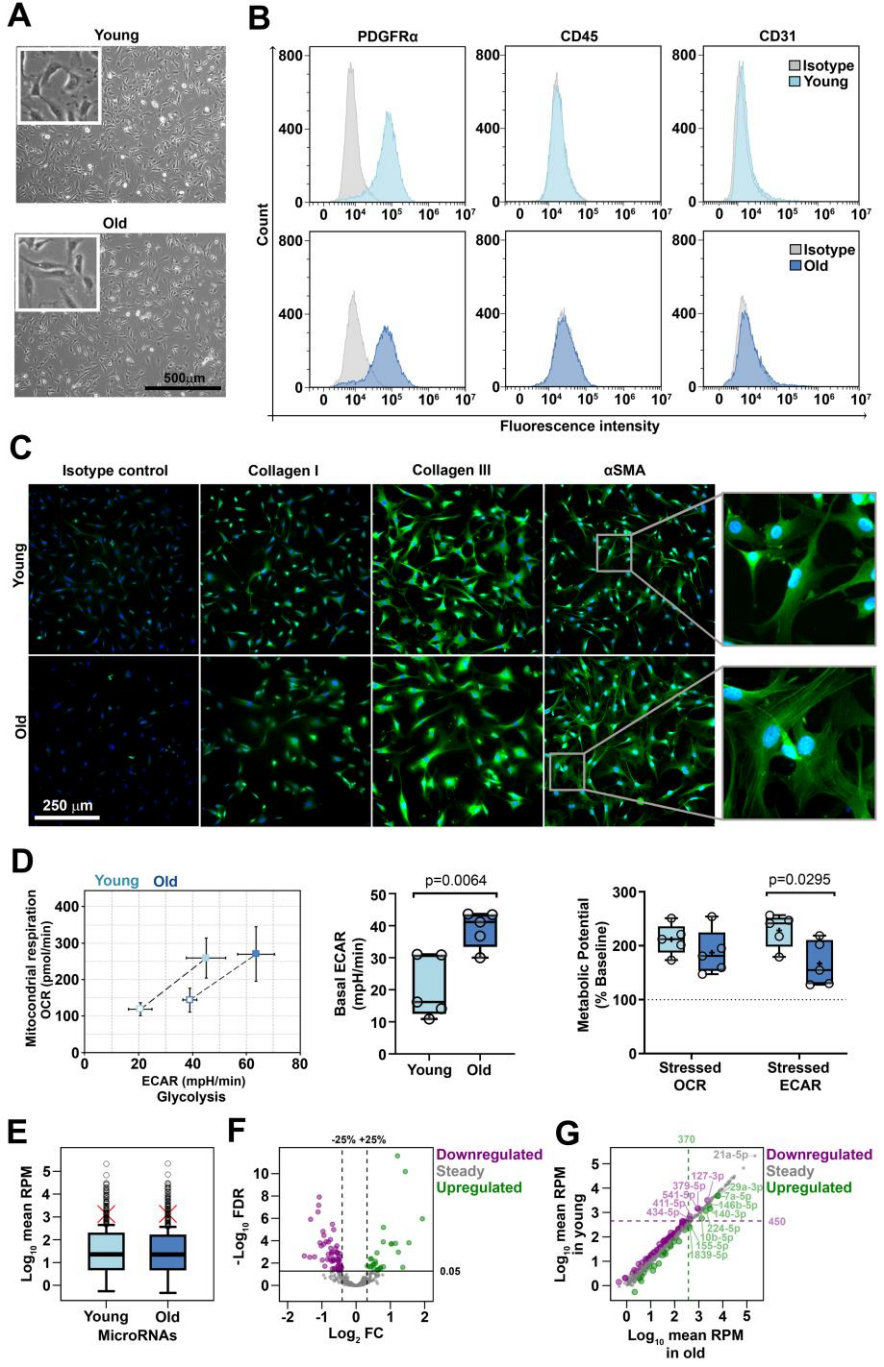
Characterization of cardiac fibroblasts derived from young and old mice (**A**) Phase-contrast microscopy of cells after 3 days in culture. No morphological differences were identified between young- and old-derived cells. (**B**) Flow cytometry analysis of young- and old-derived cells after 5 days in culture revealing the presence of PDGFRα and the absence of CD31, CD45. The histograms illustrate representative results of three experiments. (**C**) Fluorescent microscopy images illustrating the presence of αSMA, collagen I and collagen III. Note the different patterns of SMA in the two groups (inset). The pictures are representative from three experiments. (**D**) Cell energy phenotypes of young- and old-derived cardiac fibroblasts obtained by using XF Cell Energy Phenotype Report Generator. (**E**) Distribution of mean expression levels of miRNAs in young and old cardiac fibroblasts. Red crosses mark mean values. Circles mark outliers. (**F**) Volcano plot showing 530 sequenced miRNAs as steady or differentially expressed (downregulated or upregulated). MiRNAs with FDR < 0.05 were considered differentially expressed. Note that downregulated and upregulated miRNAs had a variation of at least 25% in old compared to young cardiac fibroblasts. (**G**) The expression levels of miRNAs in young *versus* old cardiac fibroblasts. Upregulated miRNAs with a mean RPM value in old cardiac fibroblasts over 370 and downregulated miRNAs with a mean RPM value in young cardiac fibroblasts over 450 were depicted as outliers. FDR, false discovery rate (p-value adjusted for multiple testing by the Benjamini-Hochberg procedure); FC, fold change (miRNA in old compared to young cardiac fibroblasts); RPM, reads per million.

Cardiac fibroblasts were isolated from young (2-3-month old) and old (16-17-month old) C57Bl/6J mice by enzymatic digestion of the cardiac ventricles followed by fibroblast selection through 5-day culture of the non-cardiomyocyte cell population in the presence of FGFβ and insulin. These fibroblast-favoring culture conditions allowed the proliferation of the cells of interest and the concomitant depletion of the contaminating cells (i.e., immune and endothelial cells). This culturing-based protocol was considered advantageous over the cell surface marker-based selection procedure, since none of the reported fibroblast markers were proved to be specific for this cell type in order to capture the whole fibroblast population. At the time of miRNA analysis, the cells were pre-confluent and spindle-shaped, with thin branched cytoplasm and no major morphological differences between young and old groups (Figure 1A). Yet, a slightly larger size of old-derived cells was noted by flow cytometry, based on light scatter parameters (Supplementary Figure 1A). Flow-cytometry analysis showed that the culture was depleted of CD45^+^ immune cells and CD31^+^ endothelial cells and most of the cells expressed PDGFRα, a well-known fibroblast marker (Figure 1B), thus confirming that the starting material was a pure culture of cardiac fibroblasts. As illustrated by immunofluorescence staining, the cells expressed type I collagen, type III collagen and smooth muscle actin (αSMA) (Figure 1C). Notably, a slight but constant difference was observed in the distribution pattern of αSMA in young- and old-derived cells: while αSMA had a diffuse distribution in young cells, it appeared organized in stress fibers in old counterparts. Having the crucial role of the oxidative stress in the induction of senescence-like phenotypes [21], the evaluation of the cellular metabolism and redox status of old- and young-derived cells were assessed as potential indicatives of a frailty phenotype. The results showed that no difference existed between young and old cells in terms of ROS production in basal conditions (Supplementary Figure 1B). However, metabolic analysis using the Seahorse bioanalyzer revealed that old-derived cells had an increased baseline glycolytic activity, in comparison to their young counterparts. Subsequently, in response to mitochondrial stressors, the anaerobic metabolic potential of old cells was reduced (Figure 1D and Supplementary Figure 1C). Together, our results suggest minimal aged-related phenotypic changes of cardiac fibroblasts in culture.

### Analysis of the miRNA profile in young and old mice-derived cardiac fibroblasts

The expression level of miRNAs in cardiac fibroblasts isolated from young (11-12 weeks old, 3 male and 3 females) and aged (72-74 weeks old, 3 males and 3 females) mice was obtained by small RNA next-generation sequencing (NGS). A total of 584 miRNAs were detected, of which 530 were analyzed for differential expression and 54 were excluded due to extremely low abundance. Global analysis of miRNA expression indicated that the miRNAs were expressed over a wide, yet similar, range in both groups, i.e., from 0.5 to more than 210,000 reads per million (RPM), with a median expression of 22.8 and 22.4 RPM in young and old groups, respectively, thus suggesting that aging did not affect the overall miRNA abundance. Around 15% of the miRNAs in both groups had extreme expression levels as compared to the whole miRNA population, being distributed above the outlier thresholds, which were at 450 RPM and 370 RPM in young and old cells, respectively (Figure 1E).

Differential expression analysis highlighted that 88 miRNAs were modified in the old group, as compared to the young group, at FDR < 0.05. Of those, 68% (60 miRNAs) were downregulated and 32% (28 miRNAs) were upregulated. Three miRNAs with up-regulated expression in old group (miR-146b-5p, miR-10b-5p, and miR497a-5p) were identified as being gender-dependent, the higher expression levels being restricted to males (data not shown). All deregulated miRNAs demonstrated at least 25% modified expression in old, relative to young, group; however, the changes were generally moderate, accounting for up to 65% decreases for downregulated miRNAs and four-time increases for upregulated ones (Figure 1F). Thus, the natural aging process did not dramatically alter the expression of miRNAs in the cardiac fibroblasts.

Several downregulated miRNAs (miR-127-3p, -379-5p, -541-5p, -411-5p, -434-5p) or upregulated miRNAs (miR-29a-3p, -7a-5p, -146b-5p, -140-3p, -224-5p, -10b-5p, -155-5p, -1839-5p) had expression levels that exceeded the outlier cutoffs. The maximal expression was 3200 RPM in young-derived cardiac fibroblasts for downregulated miRNAs and 6300 RPM in old-derived cell for upregulated ones (Figure 1G). It is noteworthy that the top 20 most abundant miRNAs were not differentially expressed in our cells. For example, miR-21a-5p, the highest expressed miRNA in cardiac fibroblasts (215 x 10^3^ RPM), had a steady expression during natural aging (Figure 1G). Other similar examples include the members of the let-7 family (let-7a, -7b, -7c, -7f, -7g, -7i), miR-10 family (miR-99, -125, -100) and miR-143. Although not affected by the aging process, these miRNAs are known to be involved in the biology of cardiac fibroblasts and/or diverse heart pathologies [22–25].

In conclusion, the miRNA profile of cardiac fibroblasts isolated from naturally aged mice revealed moderate differential expressions of 88 miRNAs, with twice more downregulated than upregulated, in the context of the overall stable abundance of total miRNA and steady expression level of highly expressed ones.

### Genomic distribution of miRNA genes with altered expression in old cardiac fibroblasts

**Figure 2:**
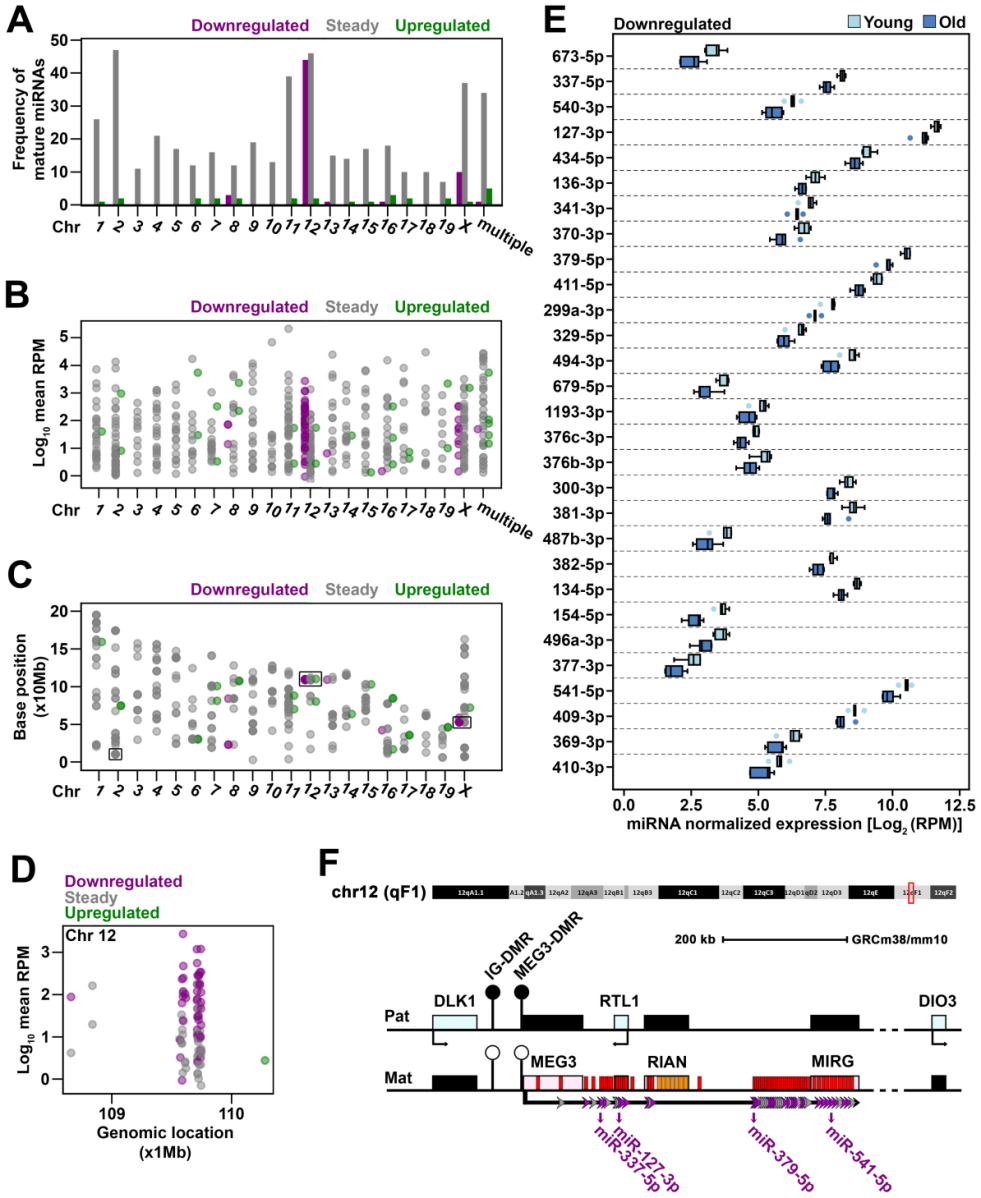
Genomic distribution of miRNAs sequenced in old cardiac fibroblasts (**A**) The frequency of mature miRNAs mapped to each mouse chromosome or to multiple chromosomes. (**B**) The expression level of mature miRNAs that mapped to each chromosome or to multiple chromosomes. (**C**) The genomic location of mature miRNAs represented by the position at which the corresponding gene begins. Mature miRNAs with multiple genomic locations were not depicted due to unknown source of expression given multiple loci. (**D**) The genomic location and expression level of miRNAs clustered on chromosome 12. (**E**) The expression of downregulated mature miRNAs mapped to Meg3-Mirg locus in old and young cardiac fibroblasts. When both -3p and -5p strands were detected, the one with greater expression was considered as representative for the respective miRNA gene. MiRNAs were ordered based on their location within the Meg3-Mirg cluster. (**F**) Schematic representation of the Meg3-Mirg locus as part of the Dlk1-Dio3 paternally imprinted locus. Meg3-Mirg locus is expressed from the maternal allele and comprises only non-coding RNAs, i.e. lncRNA (Meg3, Rian, Mirg), snoRNAs (orange bars) and miRNAs (red bars). MiRNA genes expressed in cardiac fibroblasts are illustrated as arrows: grey arrows depict steady miRNA, and purple arrows show downregulated miRNAs. MiRNAs chosen as representative for Meg3-Mirg cluster are emphasized. Additional information is shown in Supplementary Figure 2.

To identify differentially expressed miRNAs that might confidently reflect changes associated to the aging process, a screening of the genomic location of the miRNA genes was performed for all miRNAs identified in cardiac fibroblasts. Thus, the mature miRNAs were mapped to the mouse genome and the frequency of steady, upregulated and downregulated miRNAs were interrogated for each chromosome. The results showed that each chromosome harbored between 7 to 47 genes coding for steady miRNA. The upregulated miRNAs mapped on 13 out of the 20 mouse chromosomes, at one to three genes on each one. On the contrary, the downregulated miRNAs mapped on 5 chromosomes only. Of these, 44 originated from chromosome 12, while other 10 originated from chromosome X. The rest of the genes hosting for downregulated miRNAs were found having multiple locations (1 miRNA) or dispersed on chromosomes 8 (3 miRNAs), 13 (1 miRNA), and 16 (1 miRNA). As the chromosome 12 concomitantly had the second highest frequency of steady miRNAs and coded in total for 17% of all identified miRNAs in cardiac fibroblasts, it emerged as the most important source of miRNAs in these cells (Figure 2A). The expression level of miRNAs mapped on the chromosome 12 covered a similar range to the total miRNA population (Figure 2B), which thus suggested that, from a quantitative point of view, the downregulation of miRNAs on chromosome 12 was biologically relevant.

We next traced the position of the genes coding for sequenced miRNA along the length of each chromosome. To this, the first base of the sequence encoding for the mature miRNA was considered as the corresponding genomic position. The miRNA genes were generally found dispersed along the whole chromosomes, with 3 notable exceptions, in which they were grouped as clusters (Figure 2C): (i) 10 out of 48 mature miRNAs from chromosome X mapped within 6.5 kb (from miR-450b to miR-322); (ii) 24 out of 49 mature miRNAs from chromosome 2 mapped within 48 kb (miR-669f to miR-466h); and (iii) 80 out of 92 mature miRNAs from chromosome 12 mapped within 184 kb (miR-770 to miR-3072) (Figure 2D). Notably, the last cluster stood for 15% of all sequenced miRNAs (i.e., 80 out of 530) and 49% of differentially expressed mature miRNAs (i.e. 43 out of 88) in aged cells. This indicated that this cluster could be representative to the aging-associated miRNA profile and prompted us to further explore its characteristics.

The miRNA cluster on chromosome 12 was mapped to the Meg3 – Mirg locus. Meg3 – Mirg locus is part of the paternally imprinted Dlk1-Dio3 locus and is expressed as a polycistronic transcript from the positive strand of the maternal allele [26, 27]. It consists of only non-coding RNAs, i.e. 3 long-noncoding RNAs (Meg3, Rian, Mirg), several small nucleolar RNAs (C/D snoRNAs) and 57 miRNA genes (Supplementary Figure 2A). MiRNA sequencing showed that 49 miRNA genes within Meg3-Mirg locus were expressed in cardiac fibroblasts (Supplementary Figure 2A), with part of them retaining both -3p and -5p strands (Table 1), thus yielding 80 mature miRNAs. All miRNA genes within Meg3-Mirg locus were expressed at lower levels in old as compared to young group and 30 out of 49 genes were significantly modified, with a decline between 25 and 50% (Supplementary Figure 2B, 2C). As a general observation, the statistical significance was reached for the genes with higher expression levels (a median of 121.56 RPM, IQR = 343.24) as compared to those with lower expression levels (a median of 10.64 RPM, IQR = 32.67) (Supplementary Figure 2D). For accuracy, the designation “steady” was retained for the non-significantly decreased miRNAs.

**Table 1:** Strand expression of miRNAs for which both strands (-3p and -5p) were detected in cardiac fibroblasts by next-generation sequencing.

**MiRNA gene**	**Expression in young** (mean RPM*)		**Regulation in old versus young**	
	**-3p strand**	**-5p strand**	**-3p strand**	**-5p strand**
MIR1193	35.5	3.3	↓	↓
MIR127	3203.1	108.2	↓	↓
MIR136	141.4	29.8	↓	↓
MIR299a	211.3	18.6	↓	↓
MIR329	54.7	94.2	↓	↓
MIR369	78.9	23.9	↓	↓
MIR377	5.9	4.0	↓	↓
MIR379	86.0	1472.5	↓	↓
MIR382	197.4	214.2	↓	↓
MIR409	394.0	132.2	↓	↓
MIR411	142.4	688.1	↓	↓
MIR434	293.6	560.2	↓	↓
MIR540	78.7	1.4	↓	↓
MIR673	4.4	10.7	↓	↓
MIR341	121.6	3.0	↓	−
MIR370	104.4	2.2	↓	−
MIR376b	38.1	4.3	↓	−
MIR410	55.7	4.3	↓	−
MIR494	363.7	1.5	↓	−
MIR154	11.2	12.7	−	↓
MIR337	7.8	279.0	−	↓
MIR541	2.8	1464.9	−	↓
MIR679	1.6	13.5	−	↓
MIR376a	1.3	5.5	−	−
MIR380	13.7	12.4	−	−
MIR412	0.9	5.8	−	−
MIR431	13.2	29.5	−	−
MIR433	120.5	1.8	−	−
MIR485	62.7	15.9	−	−
MIR493	8.4	39.7	−	−
MIR667	11.2	8.6	−	−

Highlighted are the strands considered representative for corresponding gene expression. *RPM=reads per million.

These results suggest that the aging-associated downregulation of miRNAs within Meg3-Mirg locus is a coordinated process that may play an important role in the aging process.

### Comparative analysis of the expression level of miRNAs from Meg3-Mirg cluster in cardiac fibroblasts and cardiomyocytes during natural aging process

We next wondered whether the aging-associated downregulation of miRNAs within Meg3-Mirg locus was a specific characteristic of cardiac fibroblasts or it affected other cardiac cells as well. To address this issue, four mature miRNAs (miR-337-5p, miR-127-3p, miR-379-5p, miR-541-5p), with different locations throughout the cluster and consistent expression levels, were selected as representative for the miRNA mega-cluster within Meg3-Mirg locus to be further analyzed by qRT-PCR (Figure 2E, 2F). The expression of these miRNAs was assayed in cardiac fibroblasts and cardiomyocytes isolated from young and old animals, divided into three experimental groups: the young group (2-3 months old, n=20), the old group (16-19 months old, n=20), and the very old group (22-24 months old, n=20), with equal distribution of males and females. The animals from each group were randomly assigned for cardiac fibroblasts or cardiomyocytes isolation (n=10 per group).

**Figure 3:**
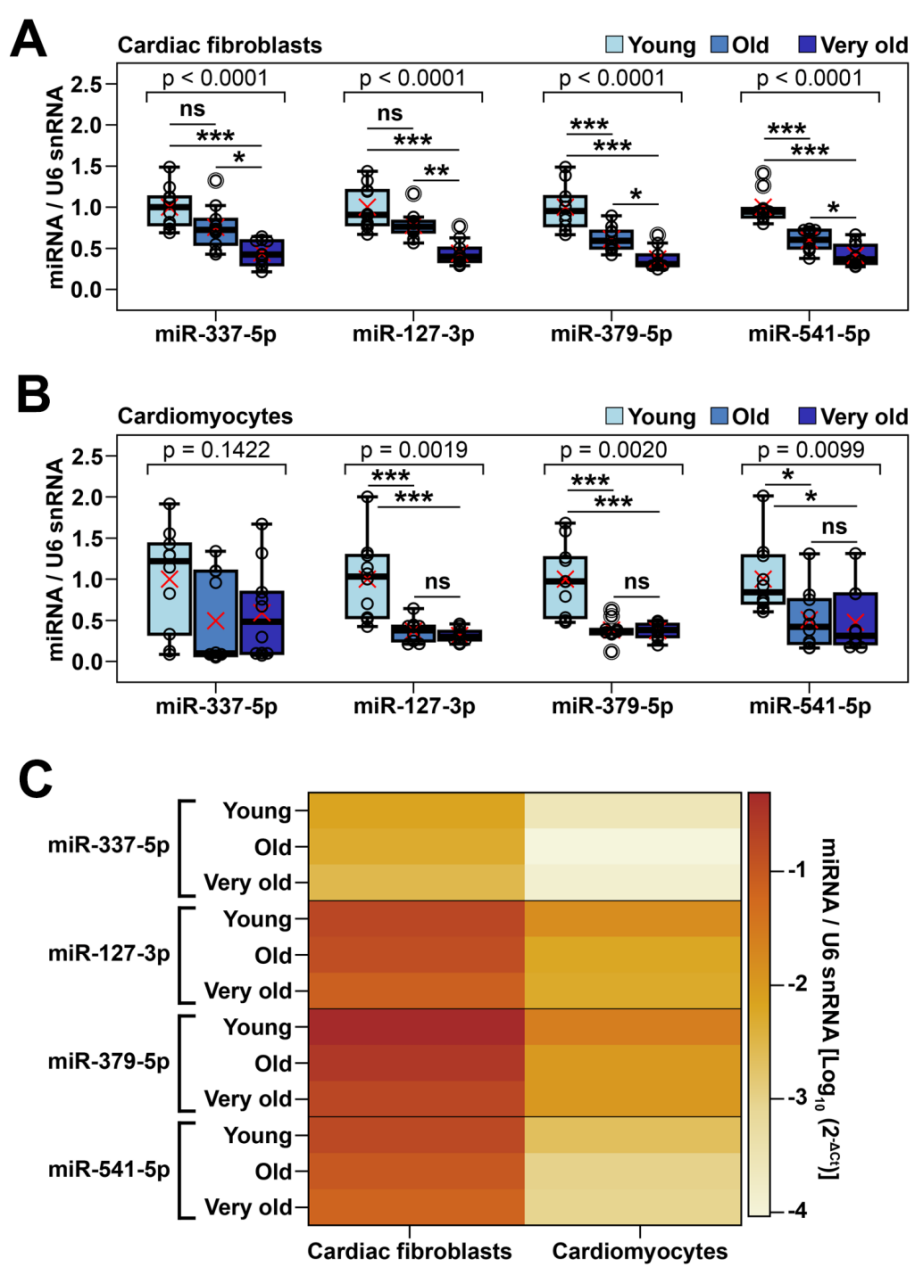
Validation of age-dependent alterations of representative miRNAs from Meg3-Mirg cluster in cardiac fibroblasts and cardiomyocytes (**A**, **B**) The expression of representative miRNAs in cardiac fibroblasts (**A**) and cardiomyocytes (**B**) in young, old and very old mice; For each miRNA, the expression is presented relative to the young group. One-way ANOVA for equal variances or Welch’s ANOVA for unequal variances, with Tukey post-hoc test for pairs; * p < 0.05, ** p < 0.01, *** p < 0.001; n=8-10/group. (**C**) Heat map showing the expression of representative miRNAs normalized to U6 snRNA in cardiac fibroblasts and cardiomyocytes harvested from young, old and very old mice. Note that cardiac fibroblasts showed higher miRNA expression than cardiomyocytes.

The qRT-PCR data showed the gradual decrease of the miRNA expression in cardiac fibroblasts from young to very old groups (22-40% decreases in old group and 56-63% decreases in very old group, respectively, relative to the young group values) (Figure 3A). These results corroborated the NGS data, thus consolidating the hypothesis that the Meg3-Mirg locus was downregulated in cardiac fibroblasts during aging. In cardiomyocytes, steep decreases (49-62%) were noted in the expression of three out of four miRNAs (miR-127, miR-379 and miR-541) in the old group, which persisted in the very old group (52-68%). MiR-337 expression had a high intragroup variability and failed to reach statistical significance (Figure 3B). Comparative analysis of the expression levels of the four miRNAs in the two cardiac cell populations showed that all miRNAs were expressed at higher levels in cardiac fibroblasts than in cardiomyocytes (Figure 3C). In accordance with NGS data, miR-127, miR-379 and miR-541 were better expressed than miR-337 in both cell types (Figure 3C). Together, these data suggested that the expression level and aging-related modulation of Meg3-Mirg cluster was not confined to cardiac fibroblasts, yet occurred in cardiomyocytes as well.

### Screening for age-dependent alteration of Meg3-Mirg cluster in multiple organs

**Figure 4:**
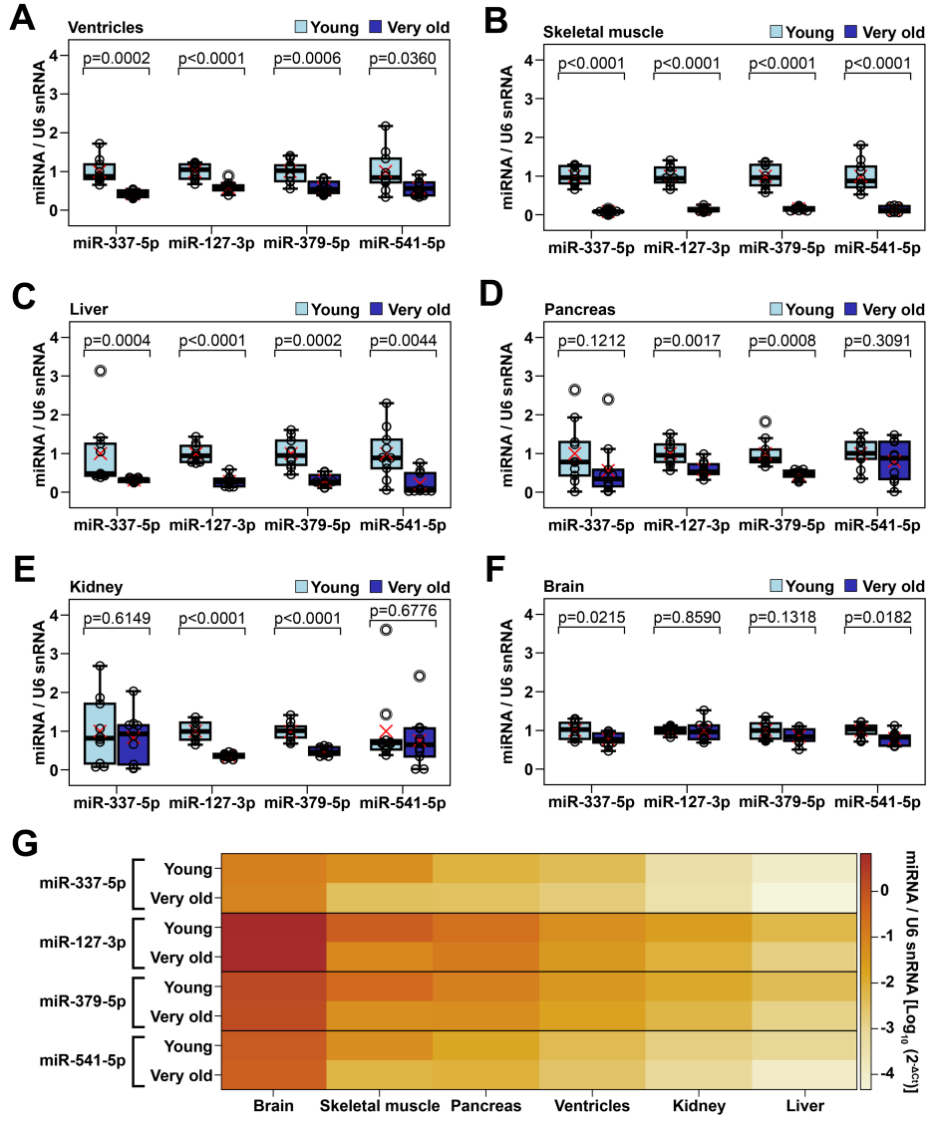
Age-dependent alteration of representative miRNAs from Meg3-Mirg cluster in several mouse organs (**A**–**F**) The expression of miRNAs in cardiac ventricle (**A**), skeletal muscle (**B**), liver (**C**), pancreas (**D**), kidney (**E**) and brain (**F**) in young and very old mice. For each miRNA, the expression is presented relative to the young group; Two-tailed two-samples T-test for equal or unequal variances; Wilcoxon two-sample test for non-normal distribution; * p < 0.05, ** p < 0.01, *** p < 0.001; n=10/group. (**G**) Heat map showing the expression of representative miRNAs normalized to U6 snRNA in mouse organs harvested from young and very old mice; Note that different organs showed variable expression of representative miRNAs (brain > skeletal muscle > pancreas > ventricles > kidney > liver).

To further document the age-induced downregulation of Meg3-Mirg cluster within the heart, the expression level of the four mature miRNAs selected as representative for the cluster was analyzed in the cardiac ventricles of young (2-3 months old, n=10) and very old animals (24-25 months old, n=10), by qRT-PCR analysis. The results showed 42-48% decreases in the expression level of the selected miRNAs in the very old group, results which were consistent with those of individual cardiac cell types (Figure 4A). Moreover, to ascertain whether Meg3-Mirg locus is transcribed as a whole unit, the expression levels of the long-noncoding RNAs within the locus (i.e., Meg3, Rian and Mirg) were also assessed in the cardiac ventricles and found to be downregulated in very old group (Supplementary Figure 3). Importantly, paternally imprinted RTL1 gene was also downregulated in aged group, however, the expressions of the flanking genes, Dlk-1 and Dio3, were not modified during aging (Supplementary Figure 3).

The above data further raised the question of whether the aging-induced Meg3-Mirg locus silencing was heart-specific or a common feature of many organs. Therefore, the expression of selected representative miRNAs was screened in other organs harvested from young and very old animals, by qRT-PCR analysis. As muscle atrophy associates with aging, skeletal muscle was included in the screening to investigate whether the age-related downregulation of selected miRNAs was a common feature of muscular organs, rather than heart restricted. To assess whether the repression was muscle specific, several non-muscular organs were also included, i.e., the liver, pancreas and kidney. Lastly, the brain, which was previously reported to maintain high transcription level of Meg3-Mirg locus during the adult life [28], was also considered in the screening.

Analysis of the skeletal muscle demonstrated that these miRNAs were almost completely silenced in aged group, with expression declines of 84-92% (Figure 4B), thus demonstrating that this silencing process was common in cardiac and non-cardiac muscle organs. A similar aging-associated downregulation pattern of the selected miRNAs was also observed in the liver, which further indicated that the Meg3-Mirg repression during aging was not muscle specific (Figure 4C). However, the pancreas and kidney showed 42-54% repressions of only two of the four miRNAs (miR-127 and miR-379) (Figure 4D, 4E), while the brain showed significant, albeit very mild (less than 25%) decreases in the other two miRNAs (miR-337 and miR-541) (Figure 4F). Noteworthy, the miRNA expression level was heterogeneous among the analyzed organs, with the highest expression levels occurring in the brain and the lowest in the liver (Figure 4G). The skeletal muscle had the second highest expression of selected miRNAs, being followed by the pancreas, cardiac ventricle, and kidney.

In conclusion, Meg3-Mirg locus was downregulated in several organs during aging, particularly in cardiac and skeletal muscle and liver. Further evidence is needed to ascertain whether the repression of Meg3-Mirg locus is a common response of all organs during aging.

### Expression of genes targeted by downregulated miRNAs from Meg3-Mirg cluster

**Figure 5:**
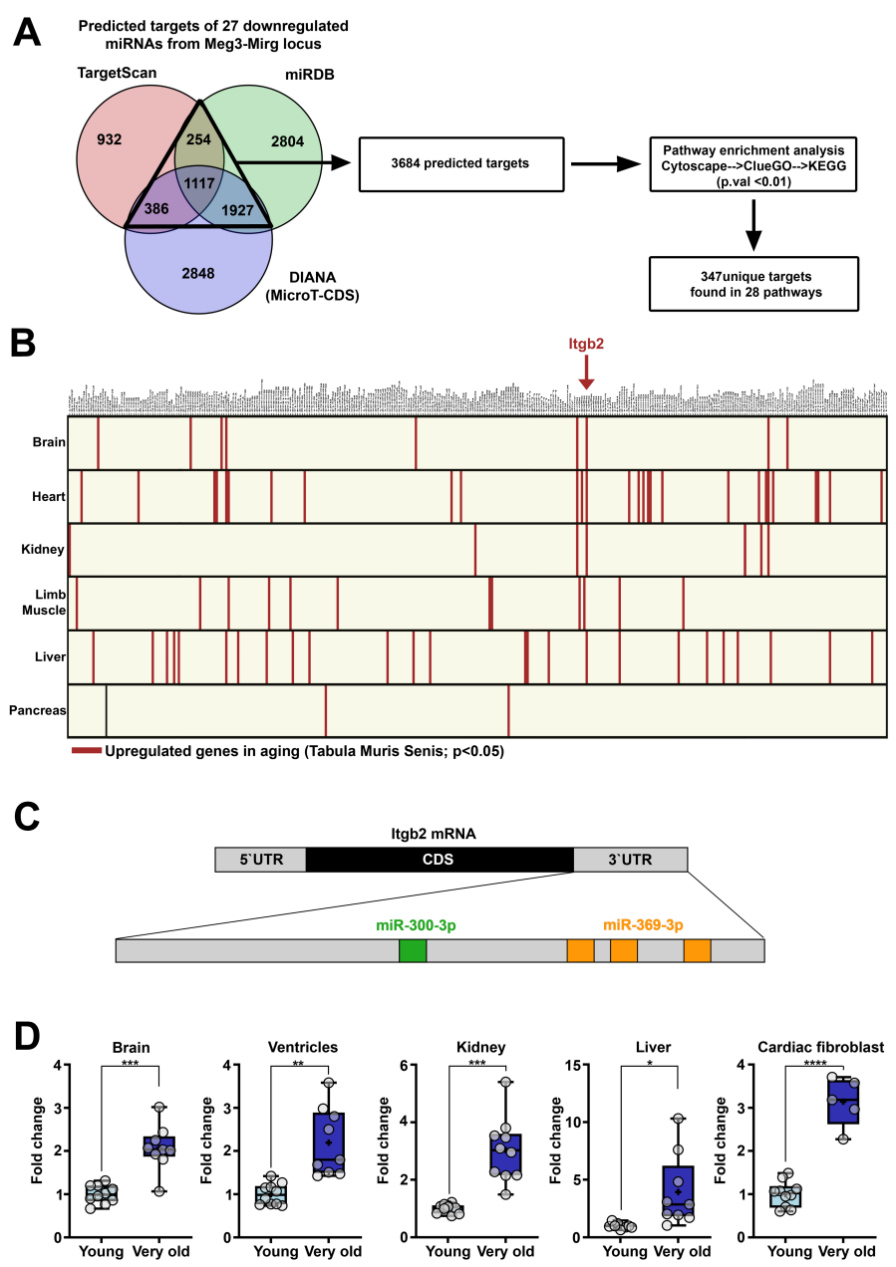
**Genes upregulated in aging which are targeted by miRNAs from Meg3-Mirg locus.** (**A**) Venn diagram illustrating the predicted targets of the 27 downregulated miRNAs. Three databases were interrogated and the overlapping targets were further used for pathway enrichment analysis, which identified 347 unique genes. (**B**) Graphical illustration of the intersection between the unique genes found in KEGG pathway and the upregulated genes identified in Tabula Muris Senis database. The upregulated genes are highlighted in each organ. Itgb2 emerged as the only gene upregulated in 4 out of 6 organs. (**C**) Illustration of the recognition sites of miRNAs from the locus targeting the 3`UTR region of Itgb2 mRNA. (**D**) The expression of the Itgb2 in the brain, ventricle, kidney, liver and cultured cardiac fibroblasts. For each organ, the expression is presented relative to the young group. Two-tailed two-samples T-test for equal or unequal variances; Wilcoxon two-sample test for non-normal distribution; * p < 0.05, ** p < 0.01, *** p < 0.001; **** p<0.0001; n=5-10/group.

The genes targeted by downregulated miRNAs in old mice were determined by combining bioinformatic analysis of the miRNA predicted targets and the publicly available databases. To this aim, 27 downregulated miRNAs (those with expression levels higher than 10 RPM) from the Meg3-Mirg locus were included in the analysis. The target genes of these miRNAs were identified as predicted by at least two of the three interrogated databases (TargetScan, miRDB and DIANA) and these target genes were further used for KEGG pathway enrichment analysis using Clue-GO (Figure 5A). KEGG pathway analysis identified 28 pathways as being enriched in the aforementioned predicted target genes (Supplementary Figure 4) with a total of 347 unique predicted targets contained in these pathways.

Aiming at identifying the aged-induced upregulated genes in the mouse organs (heart, brain, kidney, liver and limb muscle), these targets were overlapped with the upregulated genes identified in the Tabula Muris Senis database for each organ. No gene was found to be up-regulated in all 6 interrogated organs; however, Integrin beta-2 (Itgb2) was the only gene found to be overexpressed in 4 out of 6 organs (Figure 5B) and was therefore selected for further analysis. Itgb2 appeared to be part of Rap1 and Hippo signaling pathways (Supplementary Figure 4B), two evolutionarily conserved pathways involved in many cellular processes, such as cell migration and polarization, cell-cell and cell-matrix interactions and organ size control [29]. Two miRNAs within the Meg3-Mirg cluster were predicted to target the 3`UTR region of Itgb2 mRNA (Figure 5C), miR-300 (with one recognition site) and miR-369 (with three recognition sites).

To validate the upregulated expression of Itgb2 in aging, qRT-PCR analysis was performed from various organs isolated from young and very old mouse groups. The results showed that the expression of Itgb2 mRNA was upregulated in the heart, brain, kidney, pancreas, as well as in cultured cardiac fibroblasts (Figure 5D). This data suggests that Itgb2 upregulation is a common response of mouse organs during aging.

## DISCUSSION

In this study, we reported that miRNA mega-cluster within Meg3-Mirg locus was downregulated in aged cardiac fibroblasts in mice. To the best of our knowledge, this is the first report on the miRNA profile generated by NGS in cardiac fibroblasts isolated from old and young mice. The sequencing data showed that: 1) natural aging was associated with moderate differential expression of 88 miRNAs, most of them being downregulated; 2) half of the downregulated miRNAs were generated by Meg3-Mirg locus from chromosome 12.

Comparative analysis of the young- and old-derived cardiac fibroblasts revealed that the naturally aged-related phenotypic changes were unexpectedly mild. The absence of a clear phenotypic difference between young- and old-derived cells, in terms of their proliferation rate or senescence marker, makes difficult the identification of a relevant effect of the downregulated locus on the aging phenotype. In this context, the *in vitro* modulating of an individual miRNA expression will likely yield no apparent aging-relevant impact on the cellular phenotype. A more appropriate approach might be the modulation at the transcriptional level through manipulating the Meg3 promoter (by CRISPR interference technology), which would likely impact the transcription of the whole locus.

The analysis of miRNA mega-cluster within Meg3-Mirg locus was thereafter performed in other cardiac cells and organs by qRT-PCR analysis of the expression level of four miRNAs selected as representative for the cluster. Our results showed that aging was associated with the downregulation of miRNAs within Meg3-Mirg locus in cardiac fibroblasts, cardiomyocytes, heart ventricles, skeletal muscle and liver. Although the repression of the whole locus was not significant in kidney, pancreas and brain for all four selected miRNAs concomitantly, we cannot exclude the general repression of the locus, given the generally wider spread in the expression level of the not significant miRNAs. Favoring for the downregulation of the whole Meg3-Rian-Mirg locus was the qRT-PCR analysis of the long-noncoding RNAs from this locus, which showed the downregulated expressions in the cardiac ventricles in old group. Additional studies of other miRNAs and/or lncRNAs within this cluster in multiple organs will answer the question of whether the repression of Meg3-Mirg locus is universal during aging.

The imprinted Dlk1-Dio3 locus contains the largest known miRNA cluster in the mammalian genome and its organization is conserved in all mammals, thus suggesting an important role of this region. Interestingly, the post-natal expression of Dlk1-Dio3 ncRNAs is substantially downregulated in most tissues, and even more downregulated in aged tissues. Based on the data presented in this manuscript, the downward regulation of Meg3-Mirg miRNAs during aging is specific and not a passenger event, as we observed a gradual decrease of miRNA expression levels between the young, old and very old groups. This downward regulation was surprisingly reproductive, considering the very long-time span that the experiment had to accommodate (around 2 years), within an intrinsically variable setting, as a whole organism.

Our data also showed that the miRNA biogenesis was not majorly affected during natural aging in cardiac fibroblasts, as there was no difference in the overall expression of total miRNA, a result which might be explained by the cell or tissue specificity of the regulation of miRNA biogenesis during aging. Accordingly, the decline in Dicer-dependent miRNA biogenesis was previously reported during aging in preadipocytes, subcutaneous or perigonadal fat, but not in brown adipose tissue or liver [30].

MicroRNAs originate from genomic loci dispersed throughout almost the entire genome (except for Y chromosome and mitochondrial genome), being either inter- or intra-genic (intronic or exonic). MiRNA genes are generally transcribed by RNA polymerase II from their own promoters, if intergenic [31], or the promoter of the host gene, if overlap other transcription units [32]. However, there are cases of intergenic miRNA which do not demonstrate a dedicated promoter, being transcribed under one of a gene located upstream from the miRNA gene. Besides, miRNAs along relatively short genomic distances can be transcribed together, as polycistronic RNAs, which links the expression of multiple miRNAs. A such example is Meg3-Mirg locus that was demonstrated to be transcribed as a whole unit [27, 33]. Thus, Das et al reported in 2015 a ChIP-seq analysis of RNA Pol II in different mouse embryonic stem cell lines showing that the polymerase II was continuously transcribing from Meg3 to Mirg [33]. Besides, Luo et al demonstrated that the maternally expressed Meg3 gene was transcribed as a polycistron that was regulated by AFF3 at the transcriptional elongation stage [27]. This polycistronic RNA contains 57 intronic or intergenic miRNAs, along with lncRNAs and snoRNAs. This is the largest known human cluster of miRNAs, that is conserved in mammals, as it is part of the paternally imprinted Dlk1-Dio3 locus [28]. Our study revealed that half of the aging-associated deregulated miRNAs in cardiac fibroblasts originated from Meg3-Mirg locus and most of them were downregulated during aging. The mechanism governing the repression of this locus is not fully elucidated; however, several studies addressing this issue in different cells and organs pointed towards the involvement of the hypermethylation of Meg3 promoter by different DNA methyl transferases [33–35]. However, around one third of miRNA genes within this cluster, mostly those with low expression, failed to show statistically significant downregulation. Our data also showed that downregulated miRNA genes were interspaced by steady ones. A possible explanation is the differential post-transcriptional processing of discrete miRNAs within the cluster which determines the differential steady-state abundance of mature miRNAs in young cells and different downregulation rate during aging process. For example, the presence of specific sequence elements [36] or structural features [37, 38] has been shown to influence the processing rate of pri- and pre-miRNAs.

There is evidence that Meg3-Mirg locus is involved in multiple aspects of the biology of aging. For example, Meg3-Mirg locus expression was repressed in skeletal muscle during aging in mouse and humans [39], contributing to the aging-associated muscle decline. Many miRNAs generated by this locus act synergistically to control the mitochondrial biogenesis and protect against excessive ROS production, by suppression of PI3K-mTOR pathway, as well as to maintain long-term hematopoietic stem cells [40]. Besides, another study elegantly demonstrated that Meg3-Mirg locus is essential for full pluripotency of induced pluripotent stem cells [33]. Therefore, it appears that miRNAs generated from this locus are relevant to mitochondrial function and pluripotency of stem cells, which are critically declined in aging.

In order to address the functional relevance of this biological phenomenon, the expression of predicted targets of miRNAs from Meg3-Mirg locus was interrogated in Tabula Muris Senis database. The bioinformatic analysis revealed Itgb2 as being upregulated in many aged organs, thus suggesting a link between the downregulated miRNAs from Meg3-Mirg locus and Itgb2 upregulated gene expression in aging. Whether and how the simultaneous modulation of the two miRNAs for which the recognition sites were identified on Itgb2 3’-UTR region impacts the Itgb2 mRNA level in old individuals remains an issue to be addressed in our future studies.

In conclusion, this study provides evidence that aging is associated to the downregulation of miRNAs generated by the Meg3-Mirg locus in several cell types and organs. This is likely to be a common and biologically meaningful response in aging, however additional studies are required to determine whether it is universally associated with this process. Such insights may advance the understanding of the mechanisms of aging, being of general interest in the field of geroscience.

## MATERIALS AND METHODS

### Ethics statement

Investigation has been conducted in accordance with the ethical standards and according to the Declaration of Helsinki and according to national and international guidelines (the European Guidelines for animal welfare (Directive 2010/63/EU)) and has been approved by the authors' institutional review board (National Sanitary Veterinary and Food Safety Authority (no 389/22.032018)).

### Animals

C57Bl/6J mice were purchased from Jackson Laboratory and bred in the animal facility of the Institute of Cellular Biology and Pathology, under specific pathogen free conditions, in a controlled environment of 12/12 hours light/dark cycle, 21° C, 55-60% humidity, with chow and water ad libitum. A cohort of female and male C57BL/6J mice was established by maintaining the animals in normal housing and feeding conditions for up to 25 months.

### Isolation of mouse primary cardiac fibroblasts

Primary mouse cardiac fibroblasts were isolated from young (2-3-month old) and old (16-17-month old) C57Bl/6J mice using a modified version of a previously published protocol [41]. Briefly, the heart was removed from the ketamine/xylazine anesthetized mice and rinsed with ice-cold PBS. After mincing into 1-mm^3^ pieces, the tissue was placed in a sterile glass beaker and digested with 0.1% Trypsin and 100 μg/mL Collagenase type I (Sigma) in HBSS (0.137 M NaCl, 5.4 mM KCl, 0.25 mM Na_2_HPO_4_, 0.44 mM KH_2_PO_4_, 1.3 mM CaCl_2_, 1 mM MgSO_4_, 4.2 mM NaHCO_3_) in 8-ml sequential fractions, in a water bath at 37° C, under constant stirring. The first fraction was collected after 20-minute digestion, while the remaining tissue was digested in 5-min fractions until completely digested. Each fraction was mixed with 2 mL ice-cold FBS to neutralize the enzymes and kept on ice until the end of digestion. At the end, the pooled fractions were centrifuged at 400 g for 5 minutes at 4° C. The resulting pellet was resuspended in 10 mL of culture medium (DMEM/F12 supplemented with 10% FBS, 1% Penicillin-Streptomycin-Amphotericin B, 2 mM L-Glutamine, 10 ng/μL FGF, 10 μg/mL Insulin), seeded on two 60 mm × 15 mm Petri dishes and cultured in a humidified atmosphere with 5% CO_2_ at 37° C. The medium was refreshed after 24 and 72 hours. At day 5 the cells were collected for analysis.

### Characterization of cardiac fibroblast by flow cytometry

At day 5 after isolation, primary cardiac cells were analyzed by flow cytometry using antibodies against PDGFRα (BioLegend 135916), CD31 (BioLegend 102414) and CD45 (BioLegend 103106) and corresponding isotypes. The cells (5 x 1010^4 /100 μl PBS) were blocked with 5% mouse serum (30 min at 4° C) and incubated with labeled antibody (40 min at 4° C). Washed cells were analyzed in PBS with 2% FBS and 2 μg/ml propidium iodide. Samples were recorded with CytoFLEX (Backman Coulter Life Sciences, USA) and analyzed with CytExpert Software. Twenty thousand events were recorded for each condition and viable cell population was selected after eliminating PI positive cells.

### Immunocytochemical analysis of mouse cardiac fibroblast

Five days cultured cells were seeded into 8-well Chamber Slide (Nunc™ 177402) at 10^4 cells/well. After 24h, the cells were fixed in 4% paraformaldehyde for 10 min at 24° C. Washed cells were permeabilized with 0.1% Triton X-100 for 15 min, blocked in 5% normal goat serum for 30 min and stained with primary antibody (anti-αSMA, Abcam ab5694, anti-Collagen I, NovusBio NB600-408, anti-Collagen III, Abcam ab7778 and corresponding isotype control) overnight at 4° C. Washed cells were incubated with FITC-conjugated secondary antibody (Sigma Aldrich F9887) for 1h at 24° C in the dark. Nuclei were stained with 1 ug/ml Hoechst 33258 for 10 min and samples were mounted with Fluoromount-G™ Mounting Medium (Invitrogen 00-4958-02). Samples were analyzed under a Leica DMi8 microscope using Leica Application Suite X 3.3.3.16958 acquisition software.

### Isolation of cardiomyocytes

Cardiomyocytes were obtained from Langendorff-perfused hearts as previously described (Ackers-Johnson et al., 2016; Liao and Jain, 2007). Briefly, anesthetized mice were injected i.p. with 100 U/mL heparin in normal saline. The ascending aorta was cannulated and the heart was perfused at a constant flow rate of 2.5 mL/min with the following warm (37° C) buffers: 1) divalent cations chelator buffer (5 mM EDTA, 10 mM BDM, 130 mM NaCl, 5 mM KCl, 0.5 mM NaH2PO4, 10 mM HEPES, 10 mM Glucose, 10 mM Taurine) for 3 minutes; 2) perfusion buffer (10 mM BDM, 130 mM NaCl, 5 mM KCl, 0.5 mM NaH2PO4, 10 mM HEPES, 10 mM Glucose, 10 mM Taurine, 1mM MgCl2) for 3 minutes; 3) digestion buffer (perfusion buffer with 1 mg/ml Collagenase type II, Gibco 17101-015) for 10 to 15 minutes. When the heart became pale and flaccid, it was transferred into a Petri dish with 3 mL digestion buffer and gently pulled apart with forceps to liberate the cells. The resulting suspension was pipetted for 2 minutes using a wide bore tip. The enzyme was neutralized with 5 mL cold stop buffer (perfusion buffer with 5% FBS). The cell suspension was passed through a 100-μm cell strainer. Cardiomyocytes were purified by 20-min gravity sedimentation at 24° C and used for subsequent analysis.

### Seahorse XFp analysis

Metabolic activity of cultured cardiac fibroblasts was analyzed using the Cell Energy Phenotype Test Kit and the Agilent Seahorse XF HS Mini Analyzer (Seahorse Bioscience, MA, USA). One day before the assay, cells were seeded into miniplates at 2x10^5 cells/well. The following day the culture medium was replaced with the assay medium recommended by the manufacturer and the metabolic potential was determined as Oxygen Consumption Rate (OCR, as a measure of mitochondrial respiration rate) and Extracellular Acidification Rate (ECAR, as a measure of glycolysis rate) under baseline and stressed conditions.

### Evaluation of ROS production

The production of Reactive Oxygen Species (ROS) in cardiac fibroblasts was analyzed using DCFDA/H2FCFDA-Cellular ROS Assay Kit (Abcam, MA, USA) and following the manufacturer instructions for flow cytometry measurement. Briefly, 2x10^4^ cells were incubated in 200 μl culture medium containing 20 μM DCFDA for 30 min at 37° C, and immediately analyzed on flow cytometry using CytoFLEX (Backman Coulter).

### Organ harvesting and processing

The organs were harvested from mice under surgical level anesthesia using sterile conditions. The following organs were collected: the cardiac ventricle, a portion of the right liver lobe, the pancreas, the left kidney, the left quadriceps muscle and the whole brain. The organs were immediately placed in RNase-free tubes and flash-frozen in liquid nitrogen, followed by storing at -80° C, until further processing. All the harvested organs were grinded in liquid nitrogen using a mortar and pestle and kept at -80° C until further processing.

### Total RNA extraction from organs and cells

For miRNA sequencing, total RNA was extracted from PBS-washed cell pellets using miRNeasy Mini Kit (Qiagen). The samples were homogenized in 1 mL Qiazol (Qiagen) using a vortex, followed by incubation at room temperature for 5 minutes. Chloroform was added to the lysates followed by cooled centrifugation at 12,000 g for 15 minutes at 4° C. Exactly 650 μL of the upper aqueous phase was mixed with 750 μL ethanol and RNA was precipitated on a miRNeasy mini column followed by automated washing with RPE and RWT buffer in a Qiacube liquid handling robot. Finally, total RNA was eluted in 30 μL nuclease free water. Total RNA integrity was checked using the RNA 6000 Nano Assay (Agilent, CA), as well as spectrophotometric RNA quantification (Nanodrop). All samples showed RIN values between 9.7 and 10.

For miRNA expression analysis by qRT-PCR, total RNA was extracted from cell pellets or frozen organ powders. The samples were homogenized in 1 mL TRIzol® Reagent (Invitrogen) using a vortex, followed by incubation at room temperature for 5 minutes. A volume of 200 μL chloroform was added to the lysates, followed by room temperature incubation for 3 minutes and centrifugation at 12,000g for 15 minutes at 4° C. The upper aqueous phase was mixed with isopropanol and centrifuged at 12,000g for 10 minutes at 4° C. The RNA pellet was washed twice with 75% ethanol RNA, dissolved in nuclease free water and solubilized at 55° C for 10 minutes. The RNA was quantified using a spectrophotometer.

### Next-generation sequencing

Equal amounts of total RNA (1 μg) were used for small RNA library preparation using NEB Next small RNA library preparation kit (New England Biolabs, US) according to the manufacture’s declarations. Adapter-ligated libraries were amplified using barcoded Illumina reverse primers in combination with the Illumina forward primer. Libraries were pooled equimolar on the basis of DNA 1000 Bioanalyzer quantification (Agilent, CA) and gel-based size selection was performed on the pooled library to enrich for insert sizes between 18 and 36 base-pairs. Sequencing was performed on Illumina HiSeq 2500 with 50 bp single end read. Overall quality of the next-generation sequencing data was evaluated with fastQC v0.11.8 (http://www.bioinformatics.babraham.ac.uk/projects/fastqc) and multiQC v1.7 [42]. Reads from all passing samples were adapter trimmed and quality filtered using cutadapt v1.17 [43] and filtered for a minimum length of 17 nt. Mapping steps were performed with bowtie v1.2.2 [44] and miRDeep2 v2.0.1.2 [45], whereas reads were mapped first against the genomic reference grcm38_p5_mp provided by Ensembl [46] allowing for two mismatches and subsequently miRBase v22.1 [47], allowing for one mismatch. For a general RNA composition overview, non-miRNA mapped reads were mapped against RNAcentral [48] and then assigned to various RNA species of interest. Statistical analysis of preprocessed NGS data was done with R v3.6 and the packages pheatmap v1.0.12, pcaMethods v1.78 and genefilter v1.68. Differential expression analysis with edgeR v3.28 [49] used the quasi-likelihood negative binomial generalized log-linear model functions provided by the package. The independent filtering method of DESeq2 [50] was adapted for use with edgeR to remove low abundant miRNAs and thus optimize the false discovery rate (FDR) correction.

### Reverse transcription and quantitative real-time polymerase chain reaction

Mature miRNAs were revers-transcribed to complementary DNA into a multiplexed reaction using miRNA-specific looped RT primers from TaqMan® MiRNA Assays and Applied Biosystems® TaqMan® MiRNA Reverse Transcription Kit. The real-time PCR reaction was carried out using corresponding primers and TaqMan probes from TaqMan® MiRNA Assays and TaqMan™ Fast Advanced Master Mix. The TaqMan® MiRNA Assays were targeted against mature miR-127-3p (000452), miR-337-5p (475462_mat), miR-379-5p (001138), miR-541-5p (002562). TaqMan™ miRNA Control Assay U6 snRNA (001973) was used for normalization. Twenty-five ng total RNA and 0.38 μL of each looped RT primer were used in 15-μL multiplexed revers-transcription reaction, containing 1mM dNTP, 3.33 U/μL Multiscribe MuLV RT and 0.25 U/ μL RNase inhibitor. The RT was carried out on Veriti™ 96-Well Thermal Cycler, with standard cycling conditions and the following settings: reverse transcription at 16° C for 30 minutes, followed by 42° C for another 30 minutes, stop reaction step at 85° C for 5 minutes, hold step at 4° C. Finally, the cDNA was diluted to a final volume of 40 μL in nuclease free water and stored at -20° C for up to one week. The qRT-PCR was carried out with 2 μL cDNA product and 0.2 μL primers and TaqMan probe, at a final reaction volume of 10 μL, on ViiA™ 7 Real-Time PCR System. The cycling conditions were: enzyme activation step at 95° C for 20 seconds, denaturation step at 95° C for 1 second, annealing/extension at 60° C for 20 seconds, hold step at 4° C. Forty cycles of amplification were performed.

### Bioinformatic analysis

Mature miRNAs sequenced in cardiac fibroblasts were searched on miRBase 22.1 (October 2018) database (http://www.mirbase.org/) for the corresponding Mus musculus miRBase accession number and primary miRNA annotation [47]. Using UCSC Table Browser we located miRNA genes along the mouse chromosome and collected data from GRCm38/mm10 assembly, for the corresponding RefSeq annotation, chromosome number, strand of origin, transcription start site and transcription stop site. The genomic landscape of miRNAs clustered on chromosome 12 was analyzed with UCSC Genome Browser.

### Target prediction and pathway enrichment analysis

miRNA target gene were predicted from three online databases, TargetScan, miRDB and Diana (MicroT-CDS-miTG score >7). To improve the reliability of the results only the common targets from at least two databases were selected. KEGG pathway enrichment analysis was assessed using the plug-in ClueGOv2.5.7 [51] from Cytoscape v3.8.2 [52] and the criteria for grouping the genes for each term were: gene per term 4, gene % per term 5 with a kappa score of 0.4. The enrichment/depletion test for terms was set to two-sided (enrichment/depletion) based on hypergeometric test, and the corrected method for p value was Benjamini-Hochberg. The adjusted p<0.01 was considered statistically significant.

### mRNA-miRNA target interaction

The mRNAs expression in young and old mouse organs was downloaded from GEO Accession viewer, Tabula Muris Senis: Bulk sequencing (GSE132040). This project contains bulk RNA sequencing data for 17 organs from Mus musculus across the organism's life. The total number of samples is 947 and they are distributed as 2-6 samples per organ per age (1, 3, 6, 9, 12, 15, 18, 21, 24 and 27 months). The read values are provided in the file “190214_A00111_0269_AHH3J3DSXX_190214_A00111_0270_ BHHMFWDSXX.csv”. The count data was preprocessed (normalized, filtrated for low count, and log2 transformed) and further analyzed for differential expression between groups of interest (organ, age criteria) with Limma-voom from Biocoductor [53]. The imposed threshold for significance was p<0.05 and absolute value of fch >1.1. Upregulated gene resulted from RNA-seq data from each organ were intersected with the predicted targets of downregulated miRNAs found in KEGG pathway enrichment analyses.

### Real-time RT-PCR analysis

Total RNA (500 ng) extracted from tissues was revers-transcribed using the High-Capacity RNA-to-cDNA Kit (Applied Biosystems). The cDNA was diluted to a final volume of 200 μL in nuclease free water and stored at -20° C. The qRT-PCR was carried out using SYBR™ Select Master Mix (Applied Biosystems) with 3 μL cDNA product and 400 nM primer mix at a final reaction volume of 10 μL, on ViiA™ 7 Real-Time PCR System. The cycling conditions were: UNG activity step at 50° C for 2 min, enzyme activation step at 95° C for 2 min, denaturation step at 95° C for 1 sec, annealing/extension at 60° C for 30 sec, hold step at 4° C. Forty cycles of amplification were performed. Relative expression was calculated using the comparative CT method and S18 or RPL32 were used for normalization.

### Statistical analysis

Statistical analysis was performed using SAS University Edition. Statistical significance was assessed by Two-Tailed Two-samples T-test (for equal or unequal variances) or Wilcoxon two-sample test for non-normal distribution, when two groups were compared. When more than two groups were compared, One-Way ANOVA for equal variances or Welch’s ANOVA for unequal variances, with Tukey post-hoc test for pairs were used.

### Availability of data and material

The next-generation sequencing data described in this study have been deposited in NCBI's Gene Expression Omnibus (Edgar, Domrachev, and Lash, 2002) and are accessible through GEO Series accession number GSE153214 (https://www.ncbi.nlm.nih.gov/geo/query/acc.cgi?acc=GSE153214). All line art figures are accompanied by a HTML format, which allow interactive exploration of the data. Other data supporting the findings are available from the corresponding author upon reasonable request.

## Supplementary Material

Supplementary Figures
