# Supplementary material for: miRNAs generated from Meg3-Mirg locus are downregulated during aging
Source: Aging (Albany NY). 2021 Jun 22;13(12):15875–97. doi: 10.18632/aging.203208 (PMC8266327; doi:10.18632/aging.203208)
Supplement: Supplementary Figures [file aging-13-203208-s001.pdf]

SUPPLEMENTARY FIGURES

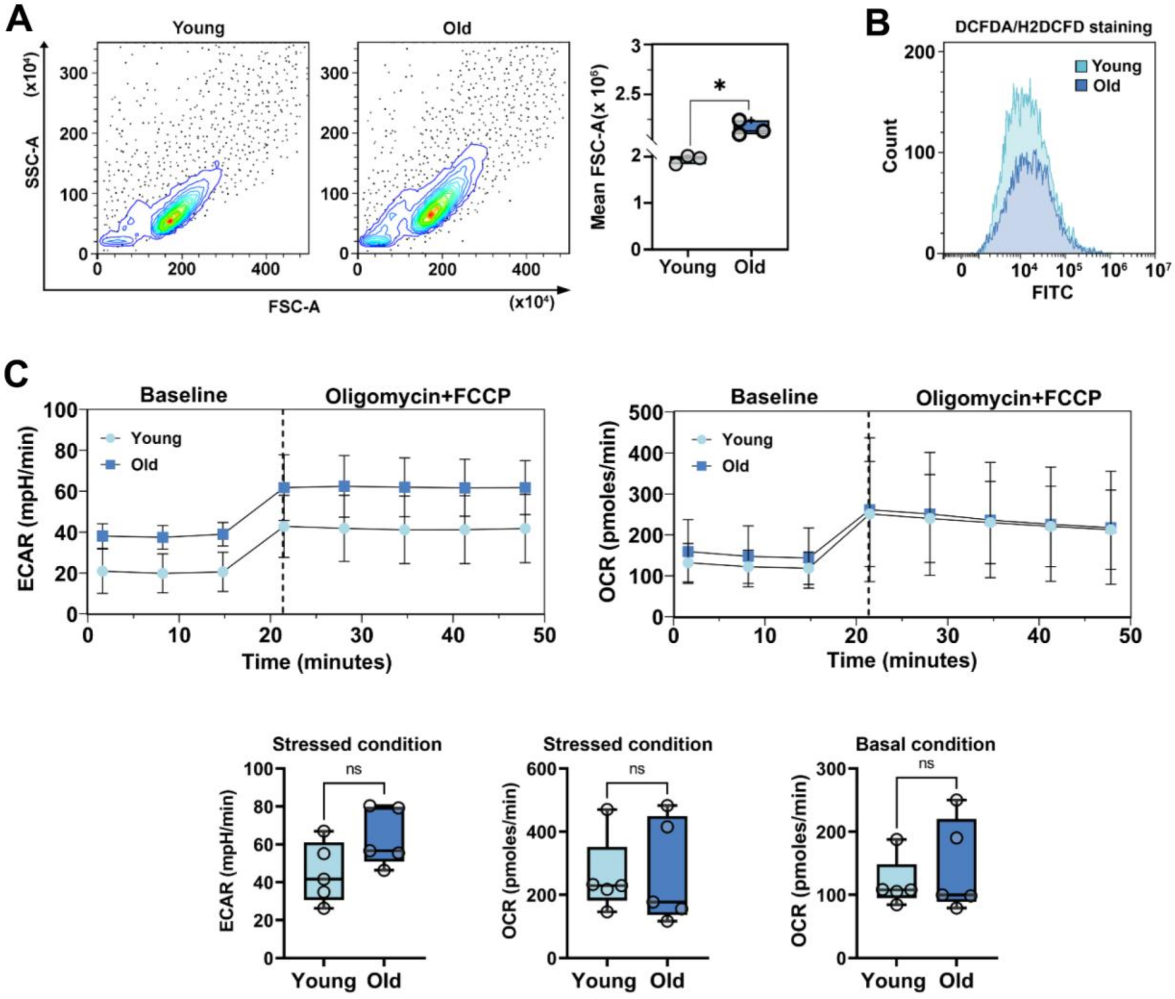

Supplementary Figure 1. Comparative characterization of mouse cardiac fibroblasts isolated from young and old mice. (**A**) Scatter plot analysis of cells illustrating the slightly larger size of old-derived cells as compared to young counterparts. (**B**) Flow-cytometry analysis of ROS production in young- and old-derived cells in basal conditions. (**C**) Metabolic analysis of cells using the Seahorse bioanalyzer.

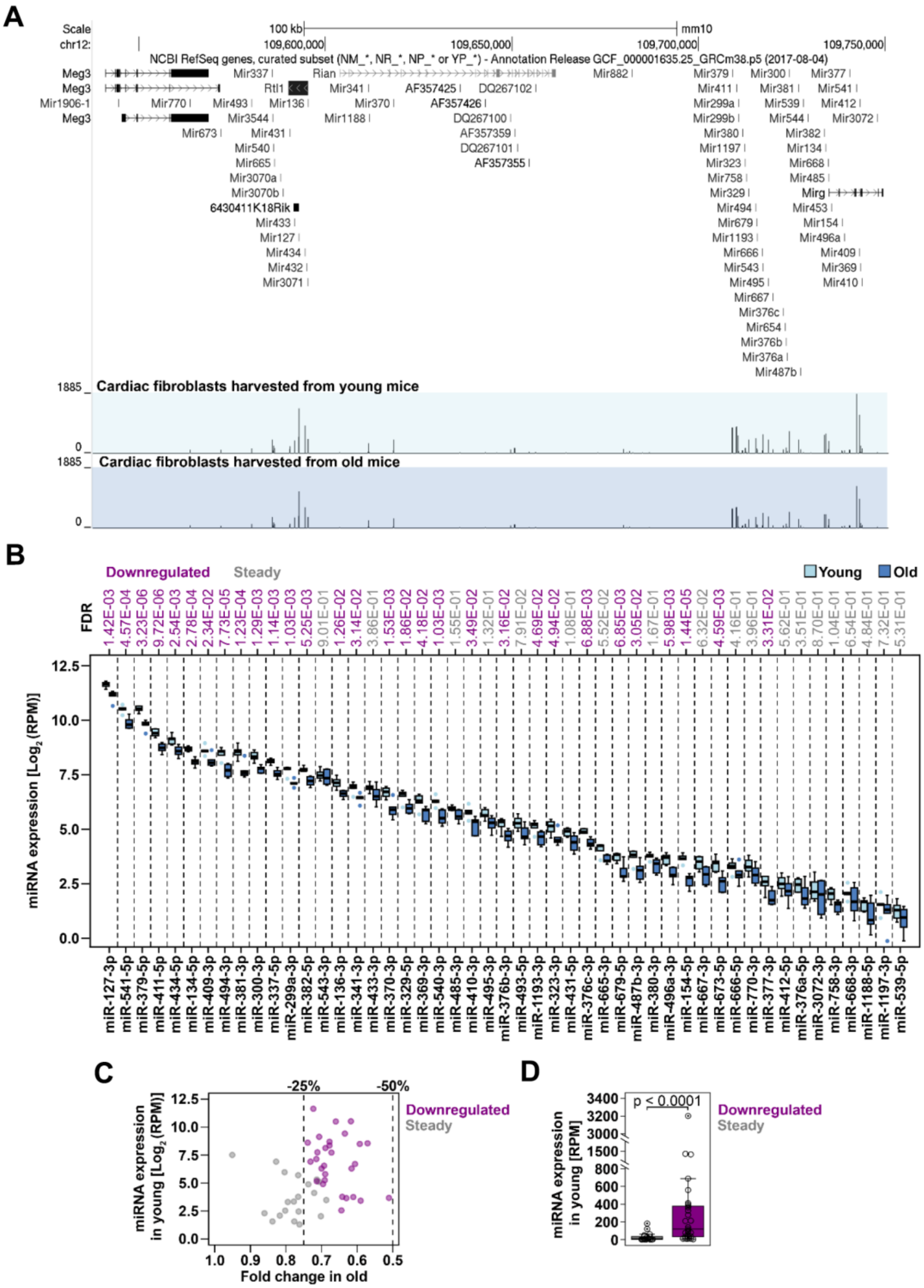

Supplementary Figure 2. Expression of miRNAs within Meg3-Mirg cluster in cardiac fibroblasts during aging. (**A**) View in the UCSC Genome Browser of Meg3-Mirg locus on mouse chromosome 12qF1 retrieved from NCBI RefSeq genes, curated subset track mapped against GRCm38/mm10 Assembly (available at www.genome.ucsc.edu). Density graphs showing the expression of miRNAs within Meg3-Mirg locus in cardiac fibroblasts harvested from young and old mice mapped against mm10 genome. (**B**) The differential expression of all miRNAs within Meg3-Mirg locus in cardiac fibroblasts harvested from old and young groups. MiRNAs are presented in descending order with respect to the mean of expression in the young group. Note that all miRNAs showed a decreasing trend in the aged group, although a few were not statistically significant. (**C**) The fold change of miRNA expression in old against the expression of the miRNA in the young group. Note that, downregulated miRNAs had generally higher expression than steady ones and the expression changed modestly (by 25-50%) in the old as compared to young group. (**D**) Scatter plots overlaid on box and whiskers plots showing higher expression in young group of those miRNAs downregulated in old group, as compared to the steady ones.

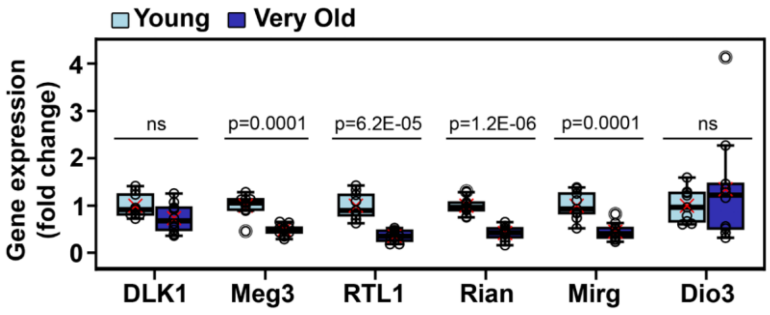

Supplementary Figure 3. qRT-PCR analysis of gene expression of coding and long-noncoding genes within Meg3-Mirg locus.

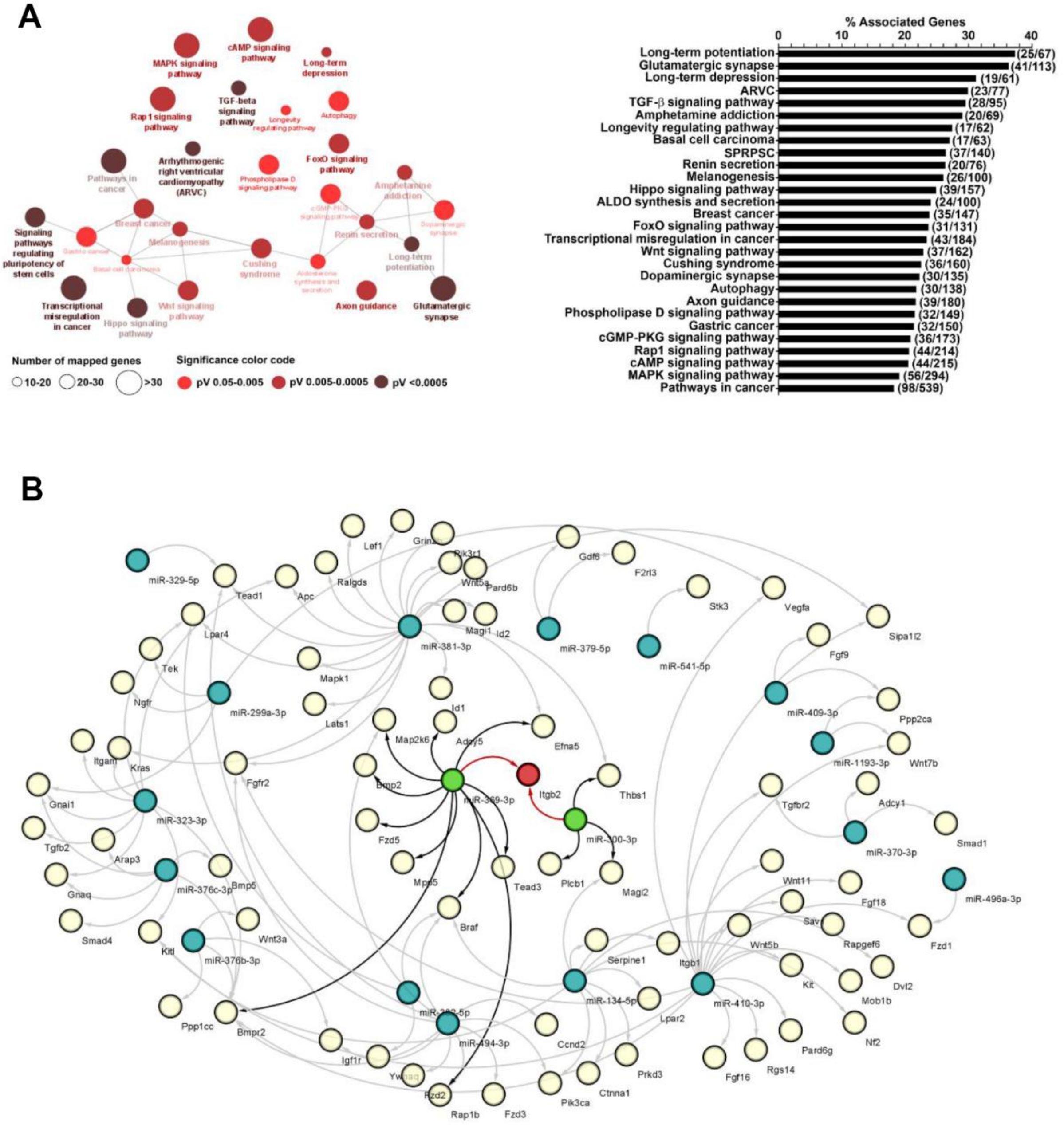

Supplementary Figure 4. (**A**) KEGG pathway enrichment analysis for the predicted target genes. Left image shows the hierarchical classification of the pathways. Node size represents the number of map genes and the node color represents the term significance. Right image illustrates the enriched pathways ordered by the percentage of the associated genes. The number of genes enriched in the pathway relative to the all genes associated to each pathway is illustrated in the brackets. (**B**) The network between the miRNAs from Meg3-Mirg locus and their associated target genes. Green nodes: miRNAs; yellow nodes: target genes. Highlighted in black are the edges from the Hippo and Rap1 signaling pathways, in which Itgb2 has been identified.
